# Evaluating Sarcopenia Prevalence in Cirrhotic Patients and Its Association With Child-Turcotte-Pugh and MELD (Model for End-Stage Liver Disease) Scores: A Cross-Sectional Study

**DOI:** 10.7759/cureus.97292

**Published:** 2025-11-19

**Authors:** Isaac I John, Modi Jainish Kamlesh Kumar, Farisha Koppath Parammal, Sheryl Elsa Abraham, Sheetal Adem, Idrees N S, Mitali B Rathod

**Affiliations:** 1 Department of General Medicine, St Stephen’s Hospital, New Delhi, IND; 2 Department of General Medicine, Dr. ND Desai Faculty of Medical Science and Research, Dharmsinh Desai University, Nadiad, IND; 3 Department of General Medicine, Karuna Medical College, Palakkad, IND; 4 Department of General Medicine, Mersey and West Lancashire Teaching Hospitals NHS Trust, Merseyside, GBR; 5 Department of General Medicine, Truworth Wellness, Hyderabad, IND; 6 Department of General Medicine, Christian Medical College Vellore, Vellore, IND; 7 Department of General Medicine, GMERS (Gujarat Medical Education and Research Society) Medical College and Hospital, Valsad, IND

**Keywords:** alcoholism, cerebrospinal fluid analysis, child-turcotte-pugh, chronic liver diseases, febrile seizures, handgrip strength, liver cirrhosis, lumbar puncture, pediatric neurology, sarcopenia

## Abstract

Background: Sarcopenia, defined as progressive loss of skeletal muscle strength and function, is a frequent but underrecognized complication of liver cirrhosis. It has been associated with increased morbidity and mortality, yet its prevalence and correlation with prognostic scores remain incompletely studied in the Indian population. The study aimed to determine the prevalence of sarcopenia in cirrhotic patients using handgrip strength and to evaluate the correlation between sarcopenia and liver disease severity, Child-Turcotte-Pugh (CTP) score, and Model for End-Stage Liver Disease (MELD) score.

Methods: A cross-sectional study was conducted among 75 adult cirrhotic patients. Handgrip strength was measured using a handheld dynamometer, and sarcopenia was defined as <27 kg in men and <16 kg in women (European Working Group on Sarcopenia in Older People 2 (EWGSOP2) criteria). Disease severity was assessed using CTP and MELD scores. Statistical analysis included Pearson correlation, with p<0.05 considered significant.

Results: The mean age of patients was 55.43 ± 11.54 years, with a male predominance of 54 (72%). Alcoholism was the leading etiology (n=45, 60%), followed by nonalcoholic steatohepatitis (NASH), hepatitis B virus (HBV), and hepatitis C virus (HCV). Mean MELD and CTP scores were 15.42 ± 6.32 and 8.42 ± 2.65, respectively, with most patients in CTP class B (60%). The overall mean handgrip strength was 26.69 ± 4.76 kg, higher in male patients than in female patients. The prevalence of sarcopenia was 42.7%. Handgrip strength declined across CTP classes (28.45 ± 5.65 kg in A, 26.87 ± 3.89 kg in B, and 21.65 ± 2.42 kg in C; p=0.001). Significant negative correlations were observed between handgrip strength and MELD (r = -0.412, p<0.001) as well as CTP (r = -0.358, p=0.01).

Conclusion: Sarcopenia is highly prevalent in cirrhosis, particularly in patients with alcohol-related disease and advanced CTP class. Handgrip strength correlates strongly with MELD and CTP scores, making it a simple and effective prognostic tool in routine practice.

## Introduction

Liver cirrhosis is a chronic liver pathology, and the hallmark clinical feature is extensive fibrosis of hepatic tissue, altered hepatic architecture, and impaired liver function [1]. It imposes a significant health burden and is one of the leading causes of morbidity and mortality globally [2]. According to the Global Burden of Disease (GBD) study 2019, globally, approximately 112 million people have compensated cirrhosis [2]. The age-standardized incidence rate in India is reported to be 30 per 100,000 population as per the GBD study 2019 [3]. In India, the most common etiology of liver cirrhosis is reported to be alcohol in 43.2% of the patients [4].

The frequent complications in liver cirrhosis are portal hypertension, hepatic encephalopathy, and malnutrition, which significantly affect the quality of life of the patients. Among these complications, sarcopenia, which causes a significant reduction in skeletal muscle mass, strength, and functionality, is emerging as a crucial factor contributing to adverse clinical outcomes. Reports indicate that sarcopenia impacts 30-70% of individuals diagnosed with cirrhosis, with the incidence of this condition escalating in correlation with its severity [5]. In a recent study conducted in India, the prevalence of sarcopenia and severe sarcopenia was estimated to be 10.09% and 6.73%, respectively [6]. These variations in sarcopenia prevalence arise from differences in diagnostic criteria, assessment tools, and study populations across global and Indian cohorts. Sarcopenia in cirrhosis patients has been associated with poor prognosis, increased risk of infections, prolonged hospitalization, diminished physical function, and higher mortality rates [7]. Despite its proven impact on prognosis, sarcopenia is frequently overlooked in clinical practice, emphasizing the need for increased awareness and the implementation of systematic diagnostic approaches.

Accurate prognostic evaluation is essential for managing liver cirrhosis, guiding treatment decisions, and predicting patient outcomes. Several scoring systems have been widely adopted for this purpose, notably the Child-Turcotte-Pugh (CTP) score and the Model for End-Stage Liver Disease (MELD) score [8]. The CTP score incorporates clinical and laboratory parameters, such as encephalopathy, ascites, serum bilirubin, albumin, and prothrombin time, providing valuable insight into hepatic functional reserve [9]. The MELD score, meanwhile, focuses primarily on objective laboratory data, including serum bilirubin, creatinine, and international normalized ratio (INR), and is currently the cornerstone for prioritizing liver transplantation [10]. Both CTP and MELD scores have gaps as they focus mainly on hepatic biochemical parameters and fail to account for extrahepatic factors such as nutritional status, muscle mass, and functional decline, which are critical determinants of prognosis in cirrhotic patients. Emerging evidence suggests a possible correlation between sarcopenia and CTP and MELD score, implying that skeletal muscle loss could reflect the severity of hepatic dysfunction and thus serve as an independent prognostic marker [11]. Hence, the present study aims to evaluate the prevalence of sarcopenia in patients with cirrhosis and explore its correlation with the severity indices provided by the CTP and MELD scoring systems, thereby contributing valuable insights for improved clinical management and prognostication of cirrhotic patients.

## Materials and methods

This was an observational cross-sectional study conducted over one year, from May 2024 to May 2025, at the Department of General Medicine, GMERS Medical College and Hospital, Valsad, Gujarat, India. The Institutional Human Ethics Committee approved the study (protocol number GMERS/MCV/IHEC/171/24), and informed consent was obtained from all participants. The study was conducted according to the Strengthening the Reporting of Observational Studies in Epidemiology (STROBE) statement [12]. 

Diagnosis criteria

While liver biopsy is considered the gold standard for diagnosing liver cirrhosis, its invasive nature may render it impractical for the study population due to patient reluctance and the associated risks in individuals with ascites and coagulopathy. Hence, the diagnosis of liver cirrhosis was established based on the following criteria. The first was a localized or generalized reduction in hepatic volume as observed on ultrasound or computed tomography of the abdomen. The second criterion was that the liver exhibits altered architecture characterized by increased or coarse echogenicity and a nodular appearance as observed on ultrasound imaging. The third criterion was that indicators of portal hypertension may be observed through clinical assessments or ultrasonographic findings, including splenomegaly, with or without the presence of ascites, and a portal vein diameter exceeding 14 mm as determined by ultrasonography. Lastly, the criteria also included indicators of hepatic cellular dysfunction, such as jaundice, decreased serum albumin levels, and prolonged prothrombin time.

Eligibility criteria

Inclusion Criteria

Patients of both genders aged ≥18 years and individuals who met the aforementioned criteria for the diagnosis of liver cirrhosis, regardless of the underlying etiology (both alcohol and viral hepatitis), and compensated and decompensated cirrhosis were included in the study.

Exclusion Criteria

Patients with other known chronic muscle-wasting conditions (e.g., malignancy, chronic kidney disease on dialysis, severe cardiac failure), acute liver failure, previous liver transplantation, and those not willing to provide consent were excluded from the study.

Sample size calculation

The sample size was calculated based on the reported prevalence of sarcopenia in 37% of Indian liver cirrhosis patients [13]. The required sample size for this cross-sectional study was calculated using the formula for estimating sample size in prevalence studies:

 $n = \frac{Z^{2} \, p \, (1 - p)}{d^{2}}$, where n = required sample size, Z = standard normal deviate (1.96 for a 95% confidence interval (CI)), p = estimated prevalence rate (based on previous studies; here, assumed to be 37% or 0.37 to provide maximum sample size), d = margin of error (desired precision, expressed as decimal; here, 11% or 0.11 chosen to approximate a total sample size around 75), giving us:

\( n = \frac{(1.96)^2 \times 0.37 \times (1 - 0.37)}{(0.11)^2} = \frac{0.89}{0.01} = 74 \)

Thus, the final recommended sample size for this study was rounded up to 75 patients.

The sample size was calculated using a 95% CI and a margin of error of 11%, based on an estimated sarcopenia prevalence of 37% in cirrhotic patients. The 95% CI for this estimate, calculated using the standard error method, was 31.5% to 53.9%, indicating a moderate-to-high burden of sarcopenia among cirrhotic patients in this cohort.

Data collection

Patient demographic details, clinical history, and detailed physical examination were recorded. Clinical parameters, including ascites, hepatic encephalopathy, presence of edema, and nutritional status, were documented. Laboratory analysis included complete blood count, serum bilirubin, serum albumin, liver enzymes (aspartate transaminase (AST), alanine transaminase (ALT), serum creatinine, International Normalized Ratio (INR), and serum electrolytes.

Assessment of sarcopenia

Sarcopenia was assessed in accordance with the European Working Group on Sarcopenia in Older People 2 (EWGSOP2) consensus guidelines, which recommend the use of muscle strength as a primary criterion. In this study, handgrip strength was used as a surrogate measure of muscle function. Measurements were taken using a calibrated handheld dynamometer, with patients seated comfortably, elbows flexed at 90^o^, and forearms resting on a flat surface. Three attempts were made for the dominant hand, with the highest value recorded. The diagnostic cut-offs applied were <27 kg for men and <16 kg for women, consistent with EWGSOP2 thresholds for low muscle strength [14]. Equipment calibration was performed prior to each testing session, and standardized measurement procedures were followed to ensure intra-observer reliability. As imaging-based assessment of muscle mass (e.g., CT or MRI) was not performed, this study identifies patients with probable sarcopenia based on reduced muscle strength.

MELD score calculation

The MELD score was calculated using the standardized formula:

\( \text{MELD score} = 3.78 \times \ln(\text{serum bilirubin (mg/dL)}) + 11.2 \times \ln(\text{INR}) + 9.57 \times \ln(\text{serum creatinine (mg/dL)}) + 6.43 \) [10].

The CTP score was calculated by evaluating five clinical parameters: encephalopathy grade, ascites severity, serum bilirubin levels, serum albumin levels, and prothrombin time (PT)/INR. Each parameter was scored from 1 to 3 points based on predefined ranges, with the total sum classifying patients into one of three categories: CTP class A (5-6 points), class B (7-9 points), or class C (10-15 points). This clinical classification provided a comprehensive assessment of hepatic functional reserve and disease severity [15].

Data analysis

IBM SPSS Statistics for Windows, version 25.0 (IBM Corp., Armonk, New York, United States) was used for the analysis. Continuous variables were presented as mean ± standard deviation (SD). Categorical variables were expressed as frequencies and percentages. Correlation analysis was performed using Pearson correlation coefficients to assess the relationships between sarcopenia and disease severity indices (CTP and MELD scores). Statistical significance was set at p <0.05.

## Results

A total of 75 patients with liver cirrhosis were included in the study. Table 1 displays the demographic and clinical characteristics of the patients. The mean age was 55.43 ± 11.54 years, with a male predominance of 72% (n=54). The most common etiology was alcoholism (n=45, 60%), followed by nonalcoholic steatohepatitis (NASH) (n=15, 20%), hepatitis B virus (HBV) (n=9, 12%), and hepatitis C virus (HCV) (n=6, 8%). The mean MELD and CTP scores were 15.42 ± 6.32 and 8.42 ± 2.65, respectively, with the majority of patients classified under CTP class B (60%). Biochemical parameters showed elevated AST and ALT levels (145.12 ± 15.34 and 154.42 ± 16.42 IU/L, respectively), while hypoalbuminemia (2.42 ± 0.24 g/dL) and a modest elevation in INR (1.56 ± 0.24) were noted. 

**Table 1:** Demographics, clinical characteristics, and biochemical parameters in study participants (N=75) MELD: Model for End-Stage Liver Disease; CTP: Child-Turcotte-Pugh

Parameters	Values
Age (years), mean±SD	55.43±11.54
Gender, n (%)	
Male	54 (72%)
Female	21 (28%)
Etiology, n (%)	
Alcoholism	45 (60%)
Non-alcoholic steatohepatitis	15 (20%)
Hepatitis B virus	9 (12%)
Hepatitis C virus	6 (8%)
Aspartate aminotransferase (IU/L), mean±SD	145.12±15.34
Alanine transaminase (IU/L), mean±SD	154.42±16.42
Total bilirubin (mg/dl), mean±SD	5.12±2.45
Albumin (g/dl), mean±SD	2.42±0.24
International normalised ratio, mean±SD	1.56±0.24
MELD score, mean±SD	15.42±6.32
CTP score, mean±SD	8.42± 2.65
CTP score classification, n (%)	
A	12 (16%)
B	45 (60%)
C	18 (24%)

The mean handgrip strength was 26.69 ±4.76 kg. Among the 75 cirrhosis patients, based on the handgrip strength of <26 kg in men and <16 kg in women, the prevalence of sarcopenia was found to be 32 (42.7%) with a CI of 31.5% to 53.9%. The mean handgrip strength in males and females was found to be 30.18±5.76 kg and 22.12±3.65 kg, respectively (Table 2).

**Table 2:** Handgrip strength among the study participants (N=75)

Variables	Values, mean±SD
Handgrip strength (Kg)	26.69 ±4.76
Handgrip strength (Kg) in male patients	30.18±5.76
Handgrip strength (Kg) in female patients	22.12±3.65

The mean handgrip strength among the CTP class A (n=12), CTP class B (n=45), and CTP class C (n=18) was found to be 28.45±5.65, 26.87±3.89, and 21.65±2.42 kg, respectively. Based on the Kruskal-Wallis test, the differences in handgrip strength across the CTP classes were found to be significant (p=0.001) (Table 3).

**Table 3:** Hand grip strength values in different Child-Pugh classes Kruskal -Wallis test; * indicates significant (p<0.05)

CTP Class	Number of Patients	Hand grip strength (Kgs), mean ±SD	P value
A	12	28.45± 5.65	0.001*
B	45	26.87±3.89	
C	18	21.65±2.42	

The Pearson correlation analysis revealed a significant negative correlation between the hand grip strength and MELD scores (Pearson correlation coefficient (r)= -0.412; p=0.001). In addition, there was a significant negative correlation between hand grip strength and CTP score (r = -0.358; p = 0.01) (Table 4).

**Table 4:** Association between handgrip strength, MELD score, and CTP score * indicates significant (p<0.05); Pearsons correlation analysis MELD: Model For End-Stage Liver Disease; CTP: Child-Turcotte-Pugh

Pearsons Correlation	Pearson's correlation coefficient	P-value
Handgrip Strength vs MELD score	-0.412	0.000*
Handgrip Strength vs CTP score	-0.358	0.01*

## Discussion

The present cross-sectional study evaluated the prevalence of sarcopenia among patients with liver cirrhosis and its association with disease severity using the CTP and MELD scoring systems. The findings demonstrate that sarcopenia is a relatively common complication in cirrhotic patients, with a prevalence of 42.7% based on handgrip strength criteria. Likewise, in a recent study by Kansal et al., the prevalence of sarcopenia in cirrhosis patients was reported to be 64% based on handgrip strength measurement [16]. In a study by Salama et al., based on the hand grip strength analysis, the prevalence of sarcopenia in HCV cirrhosis is 34.4% [17]. A study conducted in India reported a sarcopenia prevalence of 71.9% in cirrhosis patients based on the handgrip strength measurement [18]. In our study, the prevalence of sarcopenia was lower, and several reasons could explain this variation, including differences in study design, diagnostic criteria, patient selection, and the method of sarcopenia assessment. While computed tomography (CT)-based muscle mass measurement is considered the gold standard, its limited availability and cost restrict its widespread use in routine practice. In contrast, handgrip strength is a practical, noninvasive, and cost-effective tool that reflects functional impairment and has strong predictive validity for morbidity and mortality in patients with cirrhosis [19].

The present study demonstrated a mean age of 55.43 ± 11.54 years among patients with cirrhosis, with a clear male predominance (72%), aligning with prior reports of higher cirrhosis burden in men. In Atay et al.'s study, a male preponderance was observed, comprising 58.7%, and the median age of the patients was 66.5 years [20]. The most common etiology in the present study was alcoholism (60%), followed by NASH, HBV, and HCV, reflecting both lifestyle and viral contributions to liver disease. Likewise, in the study by Evuri et al., the primary cause of the chronic liver disease was due to alcoholism (59.6%) [21]. This emphasizes the continued burden of alcohol-induced hepatic injury in Indian populations and its potential link to early-onset sarcopenia through synergistic effects on nutritional deficiency, metabolic imbalance, and myotoxicity.

The mean MELD score was 15.42 ± 6.32, while the mean CTP score was 8.42 ± 2.65, with the majority (60%) classified in CTP class B, indicating intermediate disease severity. This disease severity range is particularly important from a clinical standpoint, as it represents a transitional phase where patients are no longer compensated but have not yet progressed to irreversible decompensation. Similarly, in Kenchappa et al.'s study, the mean MELD score was 15.89, and the mean CTP score was 8.5, respectively [11].

Biochemical parameters revealed elevated AST (145.12 ± 15.34 IU/L) and ALT (154.42 ± 16.42 IU/L), consistent with hepatocellular injury. A marked hypoalbuminemia (2.42 ± 0.24 g/dL) suggests impaired hepatic protein synthesis and contributes to muscle wasting. Saeki et al.'s study indicated that elevated SARC-F (Strength, Assistance in walking, Rising from a chair, Climbing stairs, and Falls) scores correlated with moderate to severe malnutrition, as determined by the controlling nutritional status score, which is derived from serum albumin levels, total lymphocyte counts, and total cholesterol levels in cirrhosis patients [22].

The INR (1.56 ± 0.24) was modestly prolonged, signifying impaired synthetic function. These abnormalities correlate with sarcopenia risk, as poor protein reserves and worsening hepatic function accelerate muscle loss. In the present study, the predominance of CTP class B patients, at 45 (60%), further highlights that sarcopenia becomes significant even before the onset of end-stage disease. In a study conducted by Sharma et al., the majority of cirrhosis patients (45%) were in the CTP class B category [23]. The review by Bozic et al. reinforces the importance of routine sarcopenia assessment in cirrhosis patients with MELD ~15 and CTP class B disease, especially in alcohol-related cases [24].

The mean handgrip strength in the current study was 26.69 ± 4.76 kg, reflecting reduced muscle function compared to healthy reference standards. Likewise, in a study by Xiao et al., the median handgrip strength among cirrhosis patients was 26.5 Kg [25]. When analyzed by gender, male patients demonstrated significantly higher strength (30.18 ± 5.76 kg) compared to females (22.12 ± 3.65 kg), consistent with normal physiological differences. However, both male and female values were lower than expected for their age groups, highlighting the presence of sarcopenia. Similarly, in Nishikawa et al.'s study, there was a marked difference in the handgrip strength among the male and female cirrhosis patients (35.5 vs 21.1 Kg) [26]. The reduced handgrip strength emphasizes the impact of cirrhosis on skeletal muscle performance. These findings support handgrip strength as a reliable and practical indicator of sarcopenia in cirrhotic patients. Integrating handgrip strength assessment into routine hepatology clinics offers a quick, low-cost tool to identify cirrhotic patients at higher risk of decompensation, enabling early nutritional and rehabilitative interventions to improve long-term outcomes.

Importantly, when analyzed according to CTP classification, patients in class A had the highest handgrip strength (28.45 ± 5.65 kg), followed by class B (26.87 ± 3.89 kg), while those in class C had markedly reduced values (21.65 ± 2.42 kg), with these differences reaching statistical significance (p=0.001). This progressive decline underscores the strong relationship between worsening hepatic dysfunction and muscle weakness, affirming sarcopenia as a clinically relevant complication in advanced cirrhosis. In a study conducted by Gaikwad et al., handgrip strength was lowest in CTP class C, followed by class B and A, highlighting the importance of the CTP score in predicting sarcopenia severity in liver cirrhosis patients [27]. 

The present study demonstrated a significant negative correlation between handgrip strength and MELD score (r = -0.412; p = 0.000), indicating that reduced muscle strength parallels worsening liver disease severity. Similarly, a significant inverse association was observed with the CTP score (r = -0.358; p = 0.01), further supporting the role of sarcopenia as a marker of hepatic decompensation. Similarly, Gaikwad et al. reported a strong negative correlation between handgrip strength and MELD scores (r= -0.394; p<0.001) and CTP scores (r= -0.606; p=0.01) [27].

These findings suggest that as hepatic reserve declines, skeletal muscle function deteriorates proportionately, reflecting the systemic catabolic effects of cirrhosis. Notably, the strength of correlation with MELD was slightly higher than with CTP, emphasizing its utility as an adjunct prognostic parameter. Such associations have been consistently reported in global studies, where sarcopenia has been independently predicted to predict mortality beyond conventional scoring systems. Thus, integrating simple measures such as handgrip strength into cirrhosis assessment may enhance risk stratification and clinical decision-making.

Limitations

This study is limited by its cross-sectional design, small sample size, and single-center setting, which may affect generalizability. Sarcopenia was assessed using handgrip strength alone, without imaging confirmation. A limited sample size may constrain the external validity (generalizability) of the findings. With smaller cohorts, the study population may not fully represent the diversity of the broader cirrhotic population in terms of age, gender distribution, etiology, nutritional status, and severity of disease. Additionally, EWGSOP2 thresholds were applied despite the lack of population-specific reference values for the Indian cohort. This study is also limited by its cross-sectional design, absence of multivariate analysis, and use of handgrip strength alone without imaging confirmation, which may introduce classification bias. Confounding variables were not adjusted for, and longitudinal outcomes were not assessed. Future multicenter studies with comprehensive sarcopenia evaluation and outcome tracking are warranted.

## Conclusions

This study demonstrates that sarcopenia is highly prevalent among patients with liver cirrhosis and shows a significant inverse correlation with both CTP and MELD scores, reflecting its association with disease severity. Recognizing sarcopenia through simple measures like handgrip strength may not only enhance prognostic accuracy but also facilitate early, targeted nutritional and rehabilitative interventions, potentially improving clinical outcomes and long-term survival in cirrhosis patients.
